# The biomechanical performance of the night-time Providence brace: experimental and finite element investigations

**DOI:** 10.1016/j.heliyon.2020.e05210

**Published:** 2020-10-17

**Authors:** Alireza Yahyaiee Bavil, Gholamreza Rouhi

**Affiliations:** Faculty of Biomedical Engineering, Amirkabir University of Technology, Tehran, Iran

**Keywords:** Biomedical engineering, Mechanical engineering, Medical imaging, Biomechanics, Biomechanical engineering, Musculoskeletal system, Mild scoliosis, Bending brace, Growth plate, Force measurement, Mechanobiology, Finite element analysis

## Abstract

The main goal of this study was to investigate the performance of a night-time Providence brace, which alters stress distribution in the growth plates and ultimately result in a reduced Cobb angle, from a biomechanical standpoint, using experimental and in-silico tools. A patient with a mild scoliosis (Cobb angle = 17) was chosen for this study. Applied forces from the Providence brace on the patient's rib cage and pelvis were measured using flexible force pads, and the measured forces were then imported to the generated FE model, and their effects on both curvature and stress distribution were observed. The measured mean forces applied by the brace were 29.4 N, 24.7 N, 22.4 N, and 37.6 N in the posterior pelvis, anterior pelvis, superior thorax, and inferior thorax, respectively, in the supine position. Results of the FE model showed that there is curvature overcorrection, and also Cobb angle was reduced from 17°, in the initial configuration, to 3.4° right after using the brace. The stress distribution, resulted from the FE model, in the patient's growth plate with the brace in the supine position, deviates from that of a scoliotic individual without the brace, and was in favor of reducing the Cobb angle. It was observed that by wearing the night time brace, unbalanced stress distribution on the lumbar vertebrae caused by the scoliotic spine's curvatures, can be somehow compensated. The method developed in this study can be employed to optimize existing scoliosis braces from the biomechanical standpoint.

## 1 Introduction

Scoliosis is one of the most common musculoskeletal disorders, which usually affects children aged from 7 to 15, and causes deformity in patient's spine that is progressive and may result in severe pulmonary and cardiovascular problems in acute cases. It is a 3-dimensional deformity of the spine and rib cage, which occurs in frontal plane and causes unwanted curves and twists in the spine [1]. The unwanted curves, caused by scoliosis, are classified into three main groups: Lumbar; Thoraco-lumbar; and Thoracic curves, depending on the area of their occurrence [2]. Two main treatments, i.e. surgery and brace treatment, are used for scoliosis, depending on Cobb angle of the curves in patient's spine. Cobb angle is a measure of curvature of spine in the frontal plane, and is used by physician to observe severity of scoliosis, and make suitable plan for the treatment.

In case of severe curves, patients may need a surgery, but in most cases with small or moderate curves, bracing is considered as the appropriate method of treatment [3]. Bracing has been the primary solution for mild and moderate scoliosis since 1946, and different kinds of braces have been introduced to date [4]. The method of treatment is based on the overcorrection of curves by pushing the spine in the opposite direction of the unwanted curves, which is expected to result in the cessation of progress of scoliotic curves [5]. The night-time braces prescriptions are growing nowadays due to their easier usage and lower time of wearing per day, in comparison to full-time braces, as well as their higher compliance with treatment [6, 7]. . These braces impose a lateral bending to the patient's spine, which results in reduction of the Cobb angle [5]. The amount of pressure needed on each site of the body, and the off-pressure areas are being estimated by the orthotist using a tentative method [6]. Charleston and Providence braces are the most common examples of night time, bending braces [7].

The performance of bending braces to prevent progression of curves was studied statistically, and their effectiveness in decelerating unwanted curves' growth was reported by many researchers to date [8]. Although the performance of bracing treatment has been statistically investigated [8, 12], the role of biomechanical factors has not been scrutinized adequately. It was assumed that stretching the concavity of the curve and compressing the convex side, along with a physiologic contracture on the convex side, may reduce the Cobb angle [13]. Due to the fact that normal stress in the growth plate (GP) of the vertebra regulates its growth over time [14, 15], it is worth to investigate the effect of employing Providence brace on the normal stress patterns of the patients' vertebrae GP, using in-silico tools, such as FEM.

Finite element method (FEM) has vast applications in orthopedic biomechanics [8, 9, 10, 11, 12, 13, 16]. For instance, in spine biomechanics, FEM can be used to investigate the effect of bending braces on the spinal column and its components, such as in vertebral bodies and intervertebral discs (IVD) [14, 15]. In 1976, forces applied from Milwaukee braces were imported into a FE model of spine and changes in curvature were reported [17]. In 2003, forces applied from a Boston brace to patient's body were measured, and imported into a FE model, and the changes occurred in the scoliotic spine curvature were investigated [18]. Gignac *et al.* have used a FE model of a scoliotic spine and found the optimized forces applied to the spinal column, which can reverse the deformed curves [19]. Wynarsky *et al.* have investigated the effect of braces on Adolescent Idiopathic Scoliosis (AIS) using optimized muscle activation configuration and a FE model [20]. In another study, a Boston brace was explicitly designed, a scoliotic FE model along with the brace was developed, and stress distribution on the spine was determined [21]. Clin *et al.* have investigated the efficiency of Charleston brace on the scoliotic spine curvature, as the first biomechanical study on the performance of bending braces [22].

## 2 Materials and methods

This study aimed to investigate the performance of the Providence brace from a biomechanical standpoint. To do this, first forces applied by the Providence brace to the ribcage and pelvis of a 13-year-old female in supine position were measured, using Pliance force pads. Then, a FE model of the scoliotic spine of the patient was developed and the measured forces were imported into the model. Finally, the stress distribution on the growth plates of vertebral bodies was calculated, and alterations in the Cobb angle were measured using FE model for model validation.

**Figure 1:**
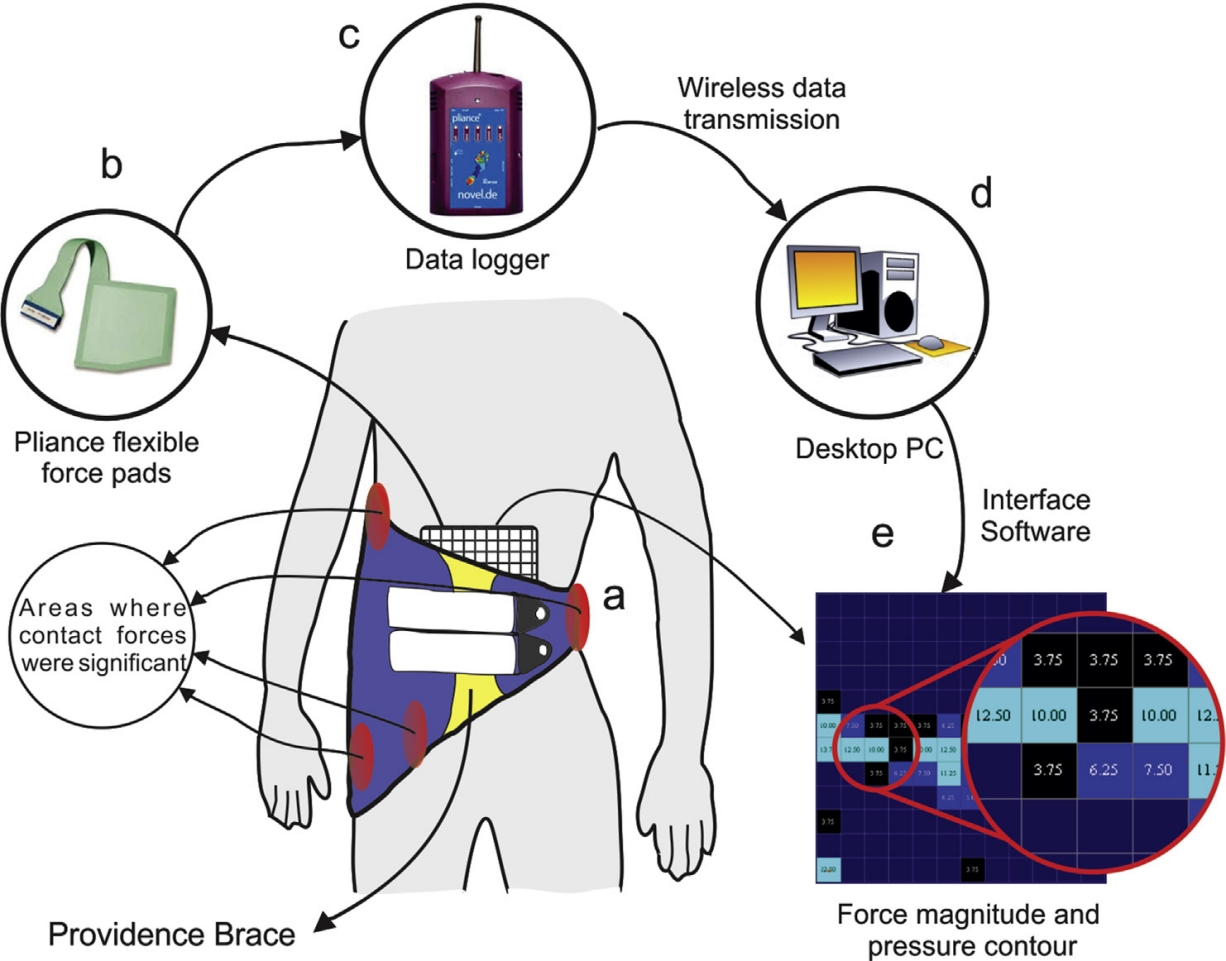
Schematic depiction of force measurement setup: (a) a patient wearing the Providence brace, the pad measures intra-abdominal pressure in supine position, and areas where contact forces were significant are highlighted in red; (b) Pliance-xf-16(-32) flexible pad; (c) Novel data logger, which records exerted forces and pressure on the pad; (d) Interface software, which reports pressure contour and force magnitude; and (e) Output data sample representing distribution of force and pressure.

This study comprises two main phases: force measurement, using Novel Pliance pads; and finite element modelling. In the experimental part, an adolescent female, aged 13, with mild scoliosis in the lumbar region (T11–L5) was selected. The test protocol was ethically approved by the Iran National Science Foundation (INSF), and legal consent was acquired from the patient's parents. The spinal indices were determined using EOS radiography images. The clinical Cobb angle was found to be 17° from L1 to L5 (Table 1). The Cobb angles related to the FE model were also measured using a frontal view of model with- and without brace in the same perspective with the EOS images. Force measurement was performed using Novel Pliance-xf- 16(-32) pads (Figure 1). The pads included a flexible sensor array, a multi-channel analyzer, a calibration device, and a software package for PC (Figure 1b, c, d). The device was first tested and calibrated using a series of known forces. Then, the forces exerted on the patient's body were measured in two different situations: (i) while the subject was wearing the brace, and the patient was in the supine position; and (ii) while the patient did not wear the brace, and was in sitting position. For the former, the forces were measured in the abdominal area, upper and lower trunk, and frontal and distal pelvis. For the latter, since the brace was not used, the force measurement was only performed in the abdominal area using abdominal belts in order to obtain the intra-abdominal pressure. For the supine position, the flexible pads were placed between the patient's skin and inner surface of the brace, then, the measurement was made and repeated, and the mean value was reported. For the latter, since the brace was not used, the force measurement was only performed in the abdominal area using abdominal belts to obtain the intra-abdominal pressure. Each measurement was conducted three times and mean value was reported.

**Table 1:** Geometric indices of the patient's spine in standing position, measured based on the EOS X-Ray photography.

Risser Sign	3
Lordosis (deg.)	41
Kyphosis (deg.)	47
Cobb Angle (deg.)	17
Deformed Region	Lumbar
Apex	L_3_
Curve side	Right-sided

**Figure 2:**
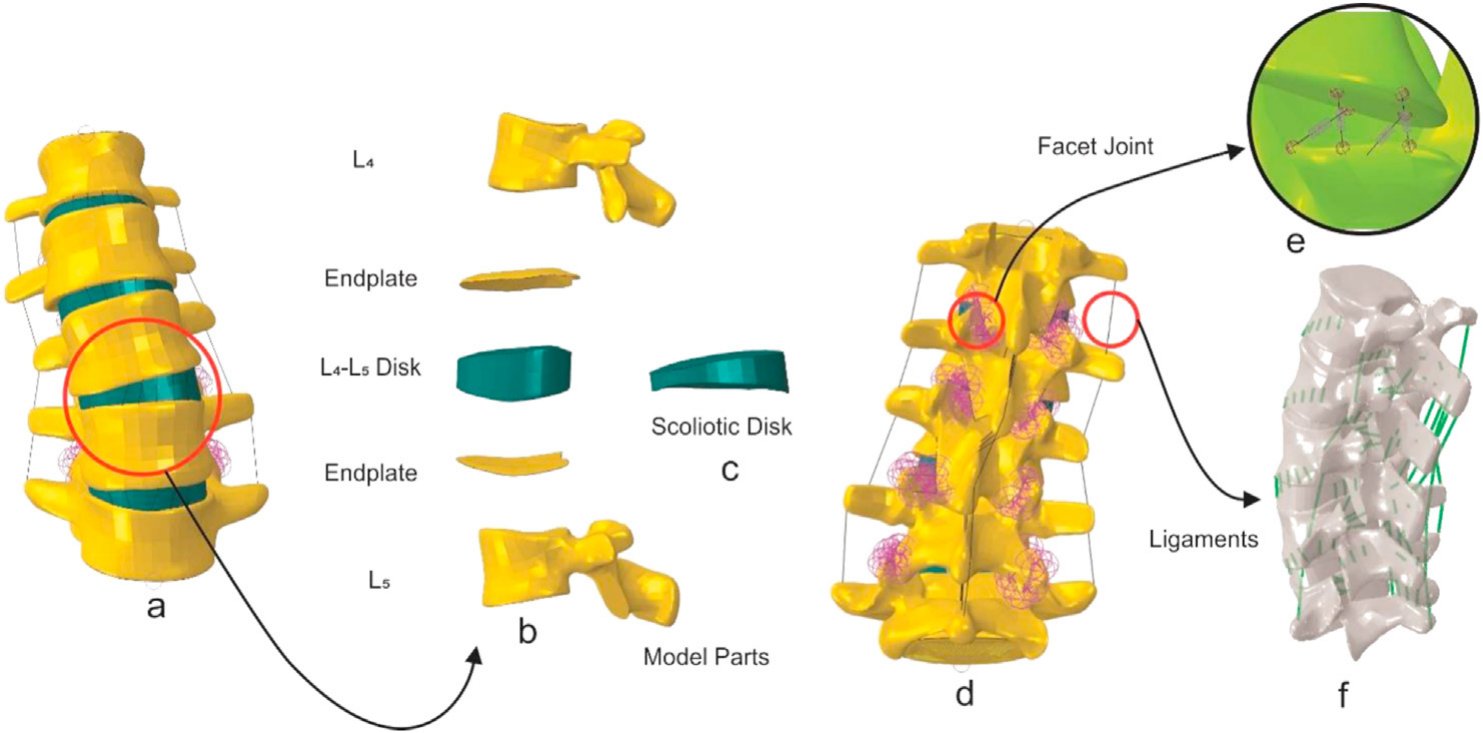
Generation of finite element model of a scoliotic spine: (a) frontal view of the scoliotic model; (b) exploded view of one-segment of scoliotic model consisting two vertebrae, two growth plates, and intervertebral disc; (c) frontal view of a deformed IV disc developed based on the patient's spinal indices; (d) posterior view of the scoliotic model; (e) facet joint modeled as four springs; (f) anterior longitudinal (ALL), posterior longitudinal (PLL), intertransverse (ITL), interspinous (ISL), supraspinous (SSL), and flavum (FL) ligaments were modeled as multi-branched tension-only springs.

The FE model was developed based on the EOS images of the patient's spine, using Mimics (Mimics 20, Materialise Inc, Leuven, Belgium) and Geomagic Design X (3D systems, North Carolina, United States) to create a CAD model of the lumbar vertebrae (Figure 2b). The intervertebral discs were modeled using SOLIDWORKS (Dassault Systèmes, Vélizy-Villacoublay, France) to fill the gaps between the vertebrae (Figure 2d). The growth plates were modeled as a 2 mm-thickness margin between vertebra and IVD (Figure 2c). The aforementioned parts were assembled using Abaqus CAE (Abaqus V 6.18, Simulia, Dassault Systèmes, and Vélizy-Villacoublay, France) (Figure 2a, b). Anterior longitudinal ligament, posterior longitudinal ligament, intertransverse ligament, interspinous ligament, supraspinous ligament, and ligament flavum were modeled as multi-branched tension-only springs (Figure 2f). Facet joints were modeled using four springs with a frictionless surface-to-surface contact [13](Figure 2e). Mesh generation was performed using 10-node tetrahedral elements, and the convergence test resulted in 424590 elements.

**Figure 3:**
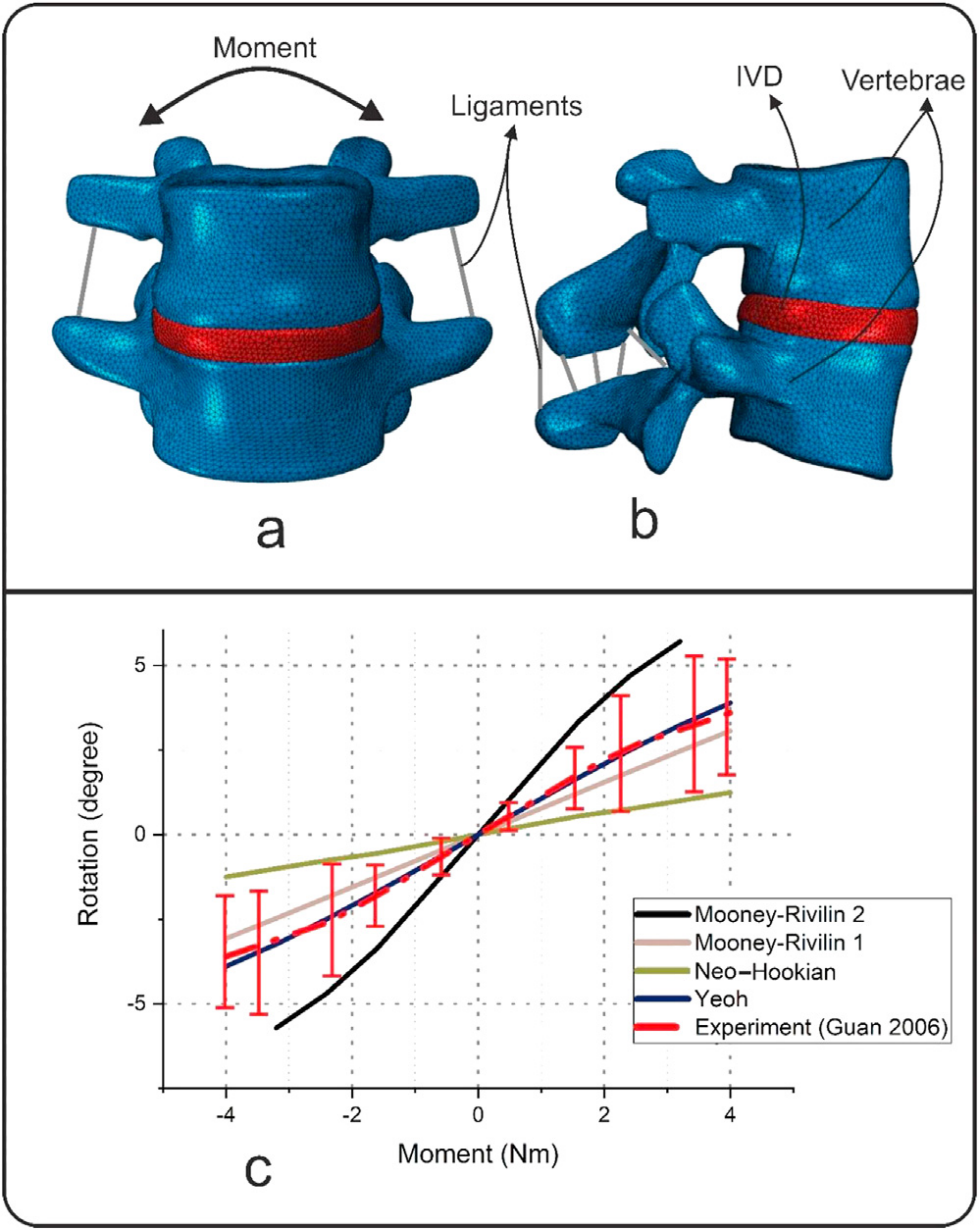
Examination of four different constitutive equations for the intervertebral disc (IVD) employing a one-segment model to find the best fit with the experimental data [27]: (a) frontal view of one-segment model consisting two vertebrae and one IVD; (b) lateral view of one-segment model; (c) results of finite element analyses of one-segment model using four different constitutive equations for the IVD, as well as an experimental work data [27]. The Yeo hyper-elastic model with the R-squared of 0.998 showed the least deviation from Guan et al.’ experimental data.

The IVDs were considered as a homogenous, isotropic, hyper-elastic material, in this study. Four different constitutive equations proposed for the IVD [23, 24, 25, 26] were examined by a one-segment model (Figure 3a, b), and the best fit with the experimental data was selected. As can be seen in Figure 3, the Yeo hyper-elastic model with an R-squared of 0.998 showed the least deviation from the reported experimental data [27]. Vertebral bodies and the posterior bony elements were modeled as a solid rigid body, and the ligaments and facet joints were assumed to be linear elastic in the range of small deformations, and their properties were assigned based on the information provided in the literature [28] (Table 2).

**Table 2:** Mechanical properties assigned to the ligaments of spinal column, and points of load application, in the FE model.

Ligament's Name	Number of fibres	Stiffness (N/mm)
Anterior Longitudinal Ligament (ALL)	5	40.5
Posterior Longitudinal Ligament (PLL)	3	25.8
Ligament Flavum (FL)	2	27.2
Intra-Spinous Ligament (ISL)	3	8.7
Supra-Spinous Ligament (SSL)	1	18
Inter-Transverse Ligament (ITL)	1	29.9
**Load Insertion Point**	**Load magnitude**	
**Posterior pelvis**	29.4	
**Anterior pelvis**	24.7	
**Superior thorax**	22.4	
**Inferior thorax**	37.6	

Based on less deformability of the ribcage, compared to cervical and lumbar spine, it was assumed to act as a rigid body, hence it was removed from the main model, and forces exerted to the ribcage were transferred to the upper surface of L1. Moreover, the pelvis and sacrum were also eliminated, and a resultant force exerted on them was applied to the lower surface of L5. Two separate loading conditions were applied to the developed FE model, which simulate the patient's two different situations, i.e. when wearing the brace and is in supine position; and without the brace and while is in sitting position. For the former, since only the lumbar spine was modelled, the forces measured by the Pliance device could not be directly applied to the model. Thus, they were replaced with a resultant force and moment on the superior surface of L1 and inferior surface of L5 using force transfer principals. Arm length for the transfer was measured between surfaces of lumbar spine and force exertion points based on anatomical indices and the brace forces were finally replaced with resultant forces and moments on the surfaces of lumbar spine. Weight of the abdominal guts and intra-abdominal pressure were applied to the anterior part of the vertebrae, as a uniform pressure. Based on the assumption that muscle forces are negligible in supine position, muscle forces were omitted. The spinous process of L3 was restricted in all directions to avoid rigid body motion, and tie constraint was considered between two adjacent surfaces of the vertebrae and IVDs. For the latter, the extensor and flexor muscles were active, thus a passive model of spine could not be used. To take this into account, two concentrated forces with the magnitude of 26.75 N and 167.23 N, measured in this study, were applied to the model to represent the forces exerted by the flexor and extensor muscles, respectively.

The FE model used in this study was partially validated using two data sets. First, one segment of the model, i.e. L_4_-L_5_, with Yeoh hyper-elastic property for the IVD, was validated based on experimental data [27](R-Square = 0.998) (Figure 3). Second, alterations in Cobb angle in the FE model was compared with clinical Cobb angle, obtained from EOS images. The clinical Cobb angle was reduced, after wearing of the brace, from 17 to 4.6°, and in the FE model, when brace forces were exerted, it was reduced from 17 to 3.4°. The model successfully simulated the patient's spine with an 4.4% error in Cob angle alterations [29].

## 3 Results

The abdominal pressures in supine position, while wearing the brace, and in sitting position, without the brace, were measured to be: 2.5 kPa and 4.32 kPa, respectively. The resultant forces of abdominal pressures in supine and sitting positions were 6.9 (±0.7) N and 12.3 (±1.1) N, respectively. The applied forces to the body caused by the Providence brace in supine position, which were measured by Novel Pliance pads were 37.6 (±2.3) N, 22.4 (±2.6) N, 24.7 (±1.7) N, and 29.4 (±1.4) N for the upper trunk, lower trunk, frontal pelvis, and distal pelvis, respectively (see Figure 1).

**Figure 4:**
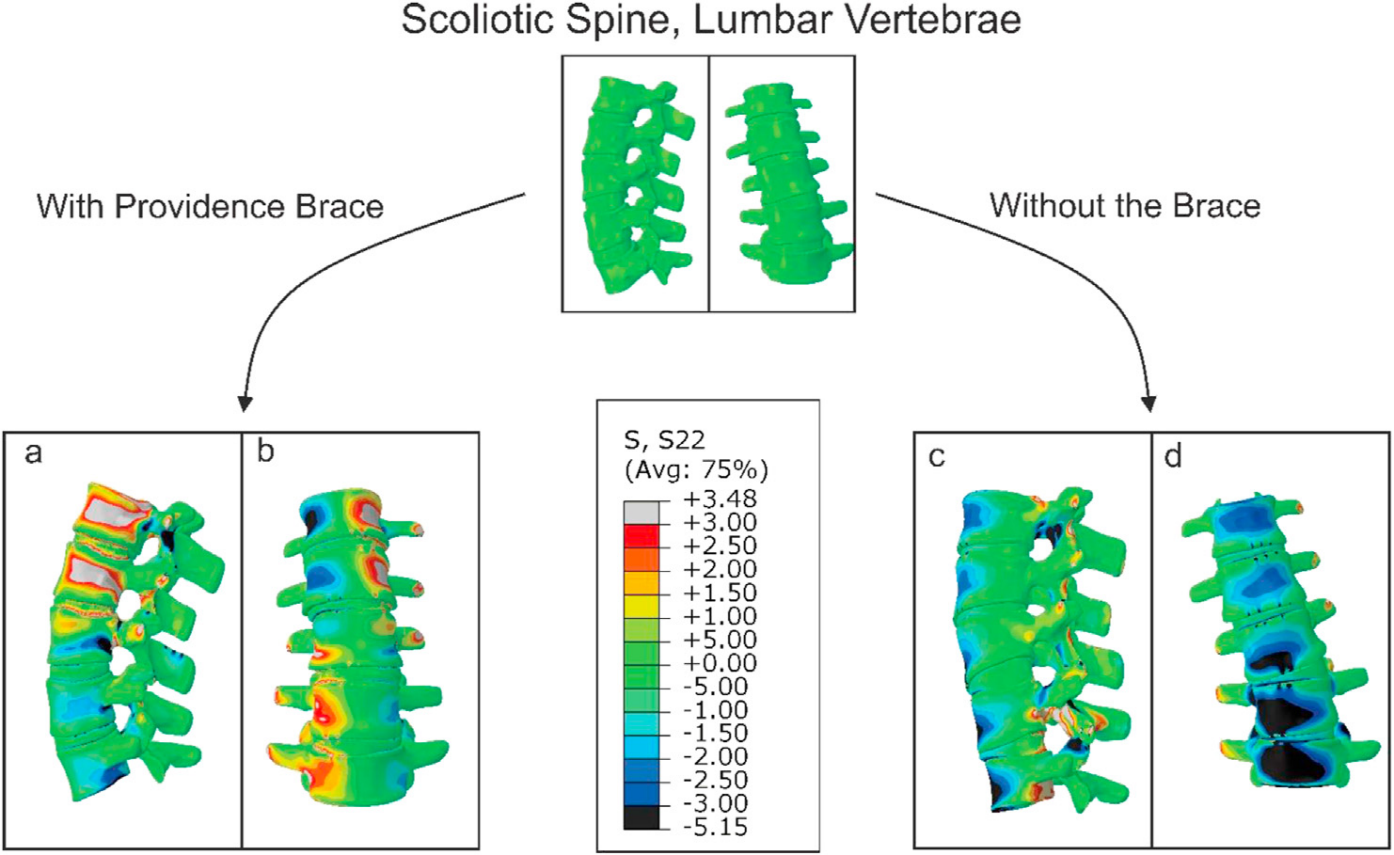
Normal stress distribution on the scoliotic lumbar vertebrae resulted from finite element analyses of the scoliotic lumbar spine: with the Providence brace and in supine position: (a) lateral view, (b) frontal view of over-corrected lumbar spine of the patient after being subjected to the brace forces, Cobb angle reduced from 17 to 3.4°; and without the brace: (c) lateral view; (d) frontal view of scoliotic lumbar spine, i.e. sitting position, the Cobb angle increased from 17 to 21°.

Deformation of the scoliotic lumbar vertebrae, and normal stress distribution on the growth plates were calculated using FE model. After importing the brace forces into the FE model, it was found that the Cobb angle measured from L1 to L5 reduced from its initial value of 17°–3.4°, without and with the brace, respectively. The clinical Cobb angle, measured based on the EOS images of the patient's trunk without and with the brace, respectively, also showed a reduction from the initial value of 17°–4.6° (Figure 4a, b).

**Figure 5:**
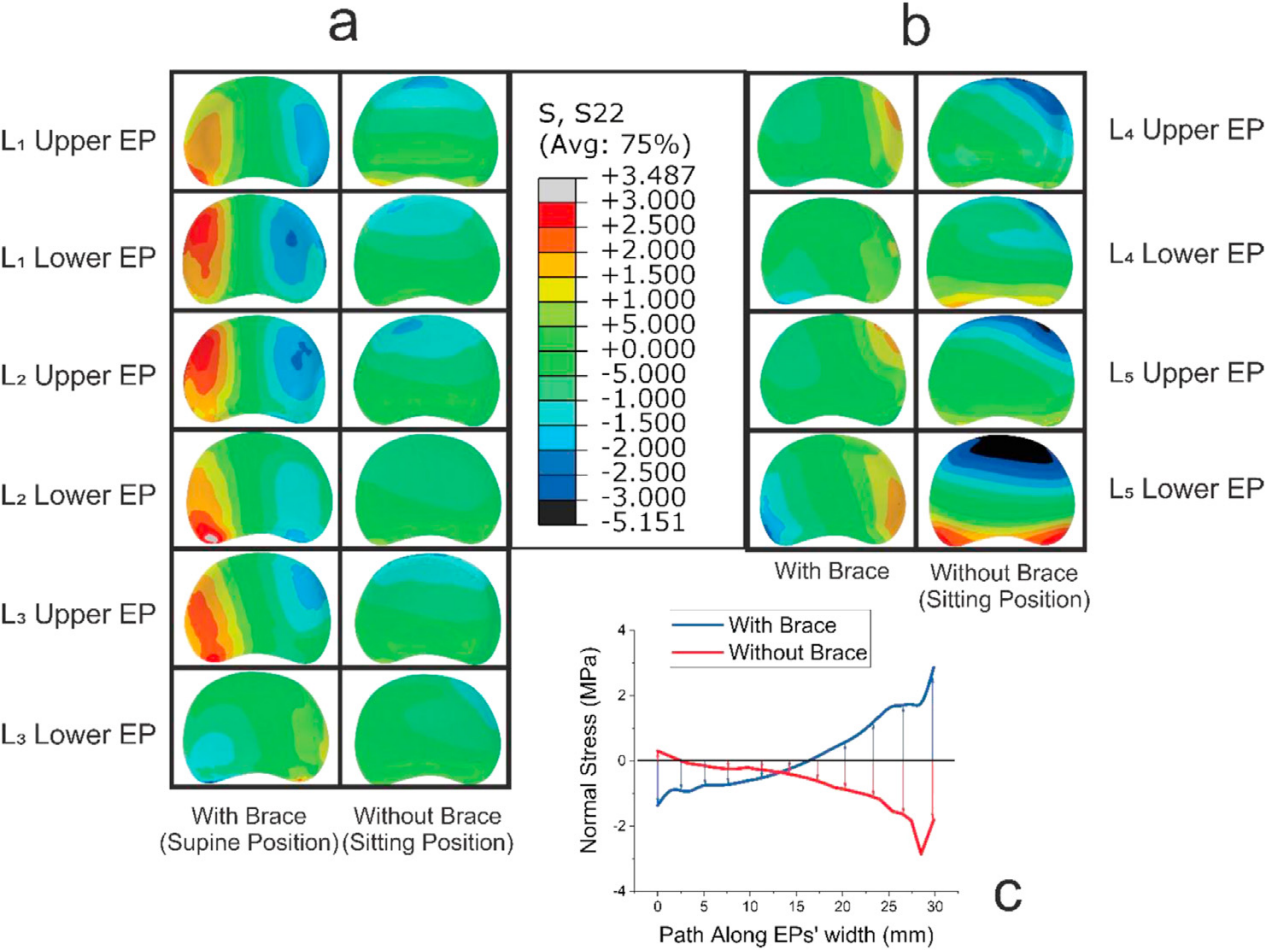
Normal stress distribution, resulted from finite element analysis, on the upper and lower growth plates of each lumbar vertebra: (a) normal stress distribution with/without the brace induced into the growth plates of L1, L2 and L3. Both tensile and compressive normal stresses were observed in the case of wearing the brace, while there was just compressive stress in the case of not wearing the brace in sitting position; (b) normal stress distribution with/without brace induced into the upper and lower growth plates of L4 and L5, which is in contrast in pattern of tensile and compressive stress regions with tensile and compressive regions in part (a); (c) Normal stress changes along upper L4 growth plates' width, which show how brace alters the stress distribution on the growth plate and thus compensates for the normal stress distribution of sitting position without brace.

In the case of using the brace and in supine position, it was found that the normal stress in both upper and lower growth plates of L1, L2, and L3 vertebrae were tensile on the left side of the growth plates, and gradually transformed into compressive on the right side of the growth plates (Figure 5a). It was also observed that in the case of wearing the brace and in supine position stress distribution caused tension on the convex side of the curvature, and compression on the concave side, which may hinder deformity's progress (Figure 5a). The stress distribution showed an opposite trend in L_4_ and L_5_ vertebrae, compared to those of L_1,_ L_2_ and L_3_ while wearing the brace and in supine position (Figure 5b). The above explained stress distributions are in favor of correcting deformity of the lumbar spine and will result in over-correction (Figure 4a, b). However, in the case of not using the brace, and in sitting position, opposite patterns of normal stress distribution were observed, compared to the case that the subject was using the brace in supine position (Figure 5), in which stress distribution caused compression on the convex side of curvature and tension on the concave side, which exacerbated the curvature by increasing the Cobb angle from $17^{{}^{\circ}}$ to $21^{{}^{\circ}}$ (Figure 4c,d).

## 4 Discussion

Bracing is a common method of treatment for scoliosis, which aims to stop the progression of scoliotic curves, and to adjust growth in the immature patients to counterbalance the asymmetrical loads on the vertebral growth plates. The method of treatment is based on the overcorrection of curves by pushing the spine in the opposite direction of the unwanted curves, which is expected to result in the cessation of progress of scoliotic curves [5]. Several disadvantages of full-time bracing, such as patient's discomfort, lack of compliance, and psychosocial factors, have made part-time bracing a felicitous method of treatment [6]. The functionality of bracing has been mostly investigated from statistical standpoint, and the role of biomechanical factors in this regard has not been extensively tackled yet. In this study, we aimed to provide a biomechanical analysis to investigate the performance of a specific type of part-time brace, i.e. the Providence brace. To do this, first applied forces to the patient's body caused by using a Providence brace, were measured using a Novel Pliance-xf-16(-32) pad. Then, a FE model of patient's lumbar spine was developed, and the measured forces in the experimental part, were imported into the model, and the normal stress distribution on the scoliotic lumbar vertebral growth plates were obtained, and alterations in the Cobb angle were observed both in presence of brace forces and without them. Finally, comparison was made between the Normal stress distributions and Cobb angle of the model with- and without the Providence brace, and in supine and sitting positions, respectively.

In the experimental section, the reaction forces were measured on the contact surfaces between the brace and the patient's body at four specific positions, where the forces are greater than all other areas, i.e. upper & lower trunk, and frontal & distal pelvis [5, 30, 31], where the pads were connected to the patient's body (see Figure 1). In the case of wearing Providence brace, the maximum force and pressure applied to the body were measured in the upper trunk region, with the values of: 37.6 N and 20 kPa, respectively, which are in agreement with some previous studies [31, 32]. The measured intra-abdominal pressure was also in close approximation with related studies [33].

**Figure 6:**
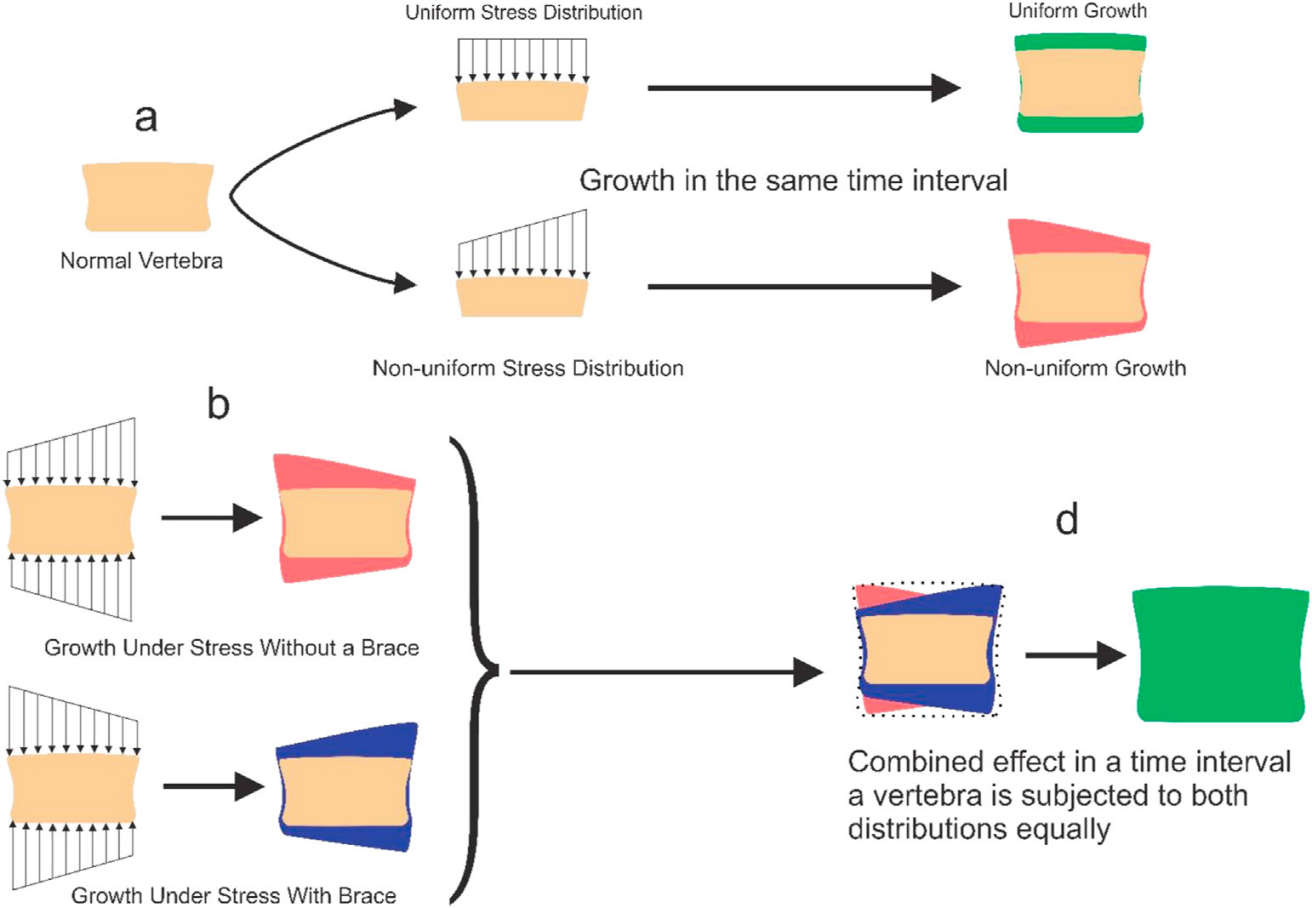
Effect of stress distribution on the longitudinal growth of vertebrae based on the Hueter-Volkmann's theory [34]: (a) A normal vertebra in a scoliosis spine, which is on the first stage of scoliosis, i.e. mild scoliosis in which the scoliotic curve is resulted from soft tissue deformations but not the vertebrae; (b) Growth of vertebrae modulated by the effect of scoliotic curve in daily activities, which results in unbalanced stress distribution and thus in a non-uniform growth pattern and effect of wearing a brace on the stress distribution, based on the simulation made in this study, which can act as a compensator to the non-uniform stress distribution due to spine deformity; and (d) Growth of a vertebra after being subjected to both with brace and without brace stress distributions equally in a time interval which supposedly is the case for patients who use the Providence part-time brace.

The scoliotic lumbar spine of the patient consists of two curves: one from L_1_-L_3_ (upper region), and the other one from L_4_-L_5_ (lower region). In the case of sitting position when no brace was used, the FE results of this study showed that normal stress distribution in the L_1_-L_3_ vertebrae was compressive on both concave- and convex sides of the vertebrae (Figure 5). But, in the case of wearing the brace in supine position, the pattern of normal stress distribution on the L_1_-L_3_ vertebrae gradually altered from tensile on the left side of the vertebrae, convex side, to compressive on the right side, concave side. Similar to the L_1_-L_3_ vertebrae, without using the brace, the FE results showed a compressive stress distribution on the left and right sides of the L_4_ and L_5_ of scoliotic vertebrae (Figure 5). Whereas, when the brace was used, the trend in the distribution of the normal stress was changed from the compressive on the left side, convex, side, to tensile on the right, i.e. concave, side of the vertebrae, respectively (Figure 5). The normal stress distributions induced in the L_1_-L_3,_ as well as in L_4_-L_5_ vertebrae, caused by using the brace can result in overcorrection (see Figure 5). Consistently, the results of FE showed that Cobb angle reduced from 17 to 4.3°, in the case of wearing the brace, which implies overcorrection (Figure 4).

various daily activities may lead to various spine configurations, which are mostly symmetrical in a normal individual, but asymmetrical in a scoliotic patient. Asymmetries in spine geometry can cause unbalanced stress distributions during daily tasks and vice-versa. Sitting position, in which most daily activities are performed, can affect spine configuration and the stress distribution within the growth plates, both in normal and scoliotic spine. Thus, investigating the stress distribution on the growth plates of vertebrae in sitting position for a scoliotic patient is crucial, considering that it can affect vertebrae's growth pattern. The FE results of sitting position, without a brace, for the scoliotic spine investigated in this research showed that there is an unbalanced stress distribution within the growth plates (Figure 5), possibly resulting in a distorted growth of vertebrae that can exacerbate the scoliosis, according to stokes, et al. 1996 [34]. For an effective bracing treatment, the brace should induce a stress distribution on the growth plates that can cause a uniform stress distribution at the end of the process, i.e. it should compensate the disturbance caused by non-uniform stress distribution resulted from a deformed spine configuration. While the patient wore the brace in supine position, and thus some parts of her body were subjected to the forces exerted by the brace, FE scoliotic model endures a stress distribution which was in contrast with the stress distribution resulted from the case that the brace was not used and the subject was in sitting position (see Figure 5a, b, 6). Based on Hueter-Volkmann's theory, from mechanobiological point of view, mechanical stress acts as a stimulus that can affect bone growth rate. Thus, it can be hypothesized that through using the night time brace, which can alter the pattern of stress distribution in the growth plates, bone growth can be compensated in order to correct the unbalanced growth of the scoliotic vertebrae (see Figure 6). Results of the previously published studies, which statistically examined the performance of night-time braces, also implied their effectiveness [13].

This work was subjected to a number of limitations. One of the limitations of this study was to use just one subject, which was partly due to the fact that a limited number of the night-time braces were prescribed for the patients in Iran to date. By increasing the number of subjects, more reliable outcomes can be gained. In regard to the FE modelling process, some simplifications were made. The intervertebral discs were assumed to be homogenous, without nucleus pulposis, and the ligaments were considered to behave linearly. Last, due to the fact that there were no experimental data on the scoliotic spine in the literature for validation, only one segment of the model was validated in our study, through comparing with a previously published experimental work [27].

## 5 Conclusions

In this study, experimental study and FE analysis were used to investigate the stress distribution within a scoliotic lumbar spine's growth plate. Another aim of this work was to find alterations occurred in Cobb angle in the lumbar spine for a patient when she worn a night time Providence brace; and when no brace was used. It was observed that a night-time brace, here Providence brace, can cause alteration in normal stress distribution within the lumbar vertebrae growth plates, which can amend the asymmetric distribution of stress, and ultimately reduce the Cobb angle (see Figure 4). It is hoped that this research can encourage other researchers to get involved in this field, and discover new aspects of spine deformity mechanisms and mechanobiology of spine deformity, and use their findings to increase the efficiency of braces, and make them more efficient in reducing the spine deformity.

## Declarations

### Author contribution statement

Alireza Yahyaiee Bavil: Conceived and designed the experiments; Performed the experiments; Analyzed and interpreted the data; Contributed reagents, materials, analysis tools or data; Wrote the paper.

Gholamreza Rouhi: Conceived and designed the experiments; Analyzed and interpreted the data; Contributed reagents, materials, analysis tools or data; Wrote the paper.

### Funding statement

This research did not receive any specific grant from funding agencies in the public, commercial, or not-for-profit sectors.

### Competing interest statement

The authors declare no conflict of interest.

### Additional information

No additional information is available for this paper.
