# ‘Corrigendum to “The biomechanical performance of the night-time Providence brace: experimental and finite element investigations” [Heliyon 6 (10) (October 2020) Article e05210]’

**DOI:** 10.1016/j.heliyon.2020.e05476

**Published:** 2020-11-18

**Authors:** Alireza Yahyaiee Bavil, Gholamreza Rouhi

**Affiliations:** Faculty of Biomedical Engineering, Amirkabir University of Technology, Tehran, Iran

In the original published version of this article, the text incorrectly cited an early conference presentation by the authors, instead of the final published paper. The correct reference is shown below. The authors apologize for the errors. Both the HTML and PDF versions of the article have been updated to correct the errors.

[9] H. Mosleh, G. Rouhi, A. Ghouchani, N. Bagheri, ‘Prediction of fracture risk of a distal femur reconstructed with bone cement: QCSRA, FEA, and in-vitro cadaver tests’, Physical and Engineering Sciences in Medicine, 43(1)(2020), 269-277.

